# Evaluation of Mean Platelet Volume in Children with Hypertension

**DOI:** 10.1155/2023/5731260

**Published:** 2023-09-21

**Authors:** Ismail Yildiz, Ozgur Kizilca

**Affiliations:** ^1^Department of Pediatrics, Faculty of Medicine, Yalova University, Yalova, Turkey; ^2^Division of Pediatric Cardiology, Department of Pediatrics, Faculty of Medicine, Tekirdağ Namık Kemal University, Tekirdağ, Turkey

## Abstract

**Background:**

Childhood hypertension, a disease with increasing prevalence, can lead to severe health problems. With the increased pressure on the vascular endothelium in hypertension, lesions in the endothelium result in endothelial activation and a process of inflammation, which causes platelet activation and in the bone marrow the release of platelet precursor cells into the peripheral blood stream. During inflammation, changes in the number and size of platelets are observed. With the release of platelet precursors into the peripheral blood stream due to platelet activation, an increase in mean platelet volume (MPV) is also seen.

**Aim:**

Our aim in this study is the evaluation of MPV changes in the hemogram of children suffering from hypertension, a condition causing severe cardiovascular problems. *Material and Methods*. This research is a descriptive retrospective cross-sectional study. It consists of a patient group diagnosed with hypertension and a control group of children presenting for routine check-ups with no diagnosed hypertension. Demographic characteristics, arterial pressure values, and hemogram parameters have been evaluated.

**Results:**

A total of 90 cases were enrolled in the study, including a patient group of 45 cases with hypertension (19 females, 26 males) and a control group of 45 cases (27 females, 18 males). The median age was 15 years in the hypertension group and 13 years in the control group. In the hypertension cases, the thickness of the carotis intima media was statistically significantly greater than in the control group (*p* < 0.001). Echocardiographic findings (IVSd, LVDd, LVPWd, IVSs, LVDs, LVPWs, and LV mass) were significantly higher in the hypertension group compared to the control group (*p* < 0.001). A statistically significant difference in platelet count and MPV values between the groups was not found (*p* = 0.151, *p* = 0.405, respectively).

**Conclusion:**

While MPV is hypothesized to be higher in hypertensive individuals, there was no statistically significant difference between the hypertensive and non-hypertensive groups in this study.

## 1. Introduction

Childhood hypertension, a disease with growing prevalence, is a significant cause of serious health problems. In childhood, depending on sex, age, and height, arterial blood pressure values below the 90^th^ percentile or, in adolescents, an arterial blood pressure below 120/80 mmHg is accepted as normotensive. Values above the 90^th^ percentile or ≥120/80 mmHg are defined as elevated arterial blood pressure and values above the 95^th^ percentile as hypertension [1–3]. In hypertension, the increased pressure on the vascular endothelium causes lesions in the endothelium, which results in endothelial activation and a process of inflammation [4–6]. Inflammation causes platelet activation and the release of platelet precursor cells into the peripheral blood [7]. The hemogram parameter measuring the size of platelets in the peripheral blood is the mean platelet volume (MPV). In inflammation, changes in the number and size of platelets are observed. With the activation of platelets and the release of platelet precursors into the peripheral blood, an increased MPV is seen. In the last years, an increasing number of studies have analyzed changes in MPV in a large range of conditions presenting with inflammation such as cardiovascular, respiratory, intestinal, and rheumatic diseases, diabetes, and cancer [8]. In addition, the MPV has been assessed as a biomarker for the prognosis of cardiovascular diseases [9].

Our aim in this study is to evaluate MPV changes in the hemogram of children with hypertension, a disease causing serious cardiovascular problems.

## 2. Materials and Methods

### 2.1. Data Collection

This research is a descriptive, retrospective, cross-sectional study. The patient group consisted of cases presenting at the Department of Pediatrics and the Pediatric Cardiology outpatient clinics with diagnosed hypertension, while the control group included children presenting for regular checkups who did not suffer from hypertension. The demographic characteristics, arterial blood pressure values, and hemogram parameters were assessed.

The inclusion criteria for the study comprised children with a diagnosis of hypertension and a complete blood count analysis was performed, no thrombocytopenia, no disease causing thrombocytopenia, and no other cardiological disease after being assessed by pediatric cardiology. However, patients with a diagnosis of thrombocytopenia or a diagnosis of a disease that would cause thrombocytopenia, splenomegaly, a known hematological disease, an additional known cardiological disease, and a complete blood count analysis during infection were excluded from the study.

#### 2.1.1. Measurement of Arterial Blood Pressure

After a resting time of at least 5 minutes, the arterial blood pressure of the cases was measured at least twice using digital measuring devices. Included in the study were individuals being followed because of hypertension and children admitted to the hospital to be diagnosed with hypertension. Hypertension in children was defined as average systolic and/or diastolic blood pressure above the 95th percentile for gender, age, and height.

#### 2.1.2. Echocardiography Evaluation

Echocardiographic examinations were performed using a 4V1c transducer with an ultrasound device (ACUSON SC2000, Siemens, Germany). Transthoracic echocardiography images were obtained in parasternal long-axis and short-axis images and apical two- and four-chamber views using standard transducer positions. The following end-diastolic and end-systolic parameters were measured in parasternal long-axis view on M-mode echocardiography: interventricular septal thickness (IVSd and IVSs), LV dimensions (LVDd and LVDs), and LV posterior wall thickness (LVPWd and LVPWs), left ventricular ejection fraction (EF) and fraction shortening (FS).

#### 2.1.3. Carotid Intima-Media Thickness Measurement

A device (ACUSON SC2000, Siemens, Germany) was used to carry out high-resolution B-mode ultrasonography of the right and left carotid arteries using a 10 MHz linear probe. The patients were examined while lying in a supine position with the head lightly extended and looking at the side opposite to the carotid artery to be checked.

#### 2.1.4. Full Blood Count

Analyses of blood samples taken in EDTA tubes were carried out with an automatic blood count device.

### 2.2. Data Analysis

Data were analyzed using an “R”-based Jamovi 1.6 package [10]. The Shapiro–Wilk test was used to test the data for normal distribution. Depending on their distribution, the data were presented as mean ± standard deviation or as median (25th- 75th percentile). The mean values of two independent groups that are normally distributed were compared by Student's *t*-test. The Mann–Whitney *U* test was used to compare differences between two independent groups that are not normally distributed. When comparing categorical groups, a chi-square test was used. Correlation analysis was performed using Pearson's or Spearman's test. A value of *p* < 0.05 was considered statistically significant.

### 2.3. Ethical Approval

This study was carried out with approval from the ethics committee for non-invasive clinical research of Tekirdağ Namık Kemal University (file number 2021.183.06.13).

## 3. Results

A total of 90 cases were included in the study: 45 cases of hypertension (19 females, 26 males) and a control group of 45 cases (27 females, 18 males). The median age in the hypertension group was 15 years (min. 8, max. 17 years), and the median age in the control group was 13 years (min. 8, max. 17 years). The characteristics of the cases are presented in Table 1.

A Holter monitoring test found 3 cases (6.7%) with pre-hypertension and 18 (40%) with hypertension. In the families of 20 hypertension cases (44.4%), a history of hypertension was present. Compared to the control group, the thickness of the carotis intima media in hypertension cases was statistically significantly greater (*p* < 0.001). In echocardiographic examination, IVSd (interventricular septal thickness at end-diastole), LVDd (left ventricular end-diastolic diameter), LVPWd (left ventricular posterior wall thickness in diastole), IVSs (interventricular septal thickness at end-systole), LVDs (left ventricular systolic diameter), LVPWs (left ventricular posterior wall thickness in systole), and LV mass (left ventricular mass) were statistically significantly higher in the hypertension cases than in the control group (*p* < 0.001). The thickness of the carotis intima and echocardiographic results of the cases are presented in Table 2.

When comparing the hemogram parameters between cases and the control group, erythrocyte count, hemoglobin and hematocrit values in the hypertension cases were found to be statistically significantly higher (*p* = 0.009, 0.032, 0.012 respectively). No statistically significant difference was found in MPV values between the groups (*p* = 0.405), which had been the aim of the study. The full blood count values of the cases are presented in Table 3.

A positive correlation was found between the thickness of the carotis intima media and weight, height, BMI, systolic arterial pressure, and diastolic arterial pressure (correlation coefficients 0.532, 0.382, 0.475, 0.587, 0.475, respectively; *p* < 0.001).

A positive correlation was found between systolic arterial pressure and IVSd, LVDd, LVPWd, IVSs, LVDs, LVPWs, and LV mass (correlation coefficients 0.632, 0.516, 0.539, 0.488, 0.499, 0.536, 0.655, respectively; *p* < 0.001).

A positive correlation was found between diastolic arterial pressure and IVSd, LVDd, LVPWd, IVSs, LVDs, LVPWs, and LV mass (correlation coefficients 0.519, 0.397, 0.504, 0.436, 0.493, 0.494, 0.531, respectively; *p* < 0.001).

For the MPV values, a positive correlation was found with PDW (*r* = 0.917, *p* < 0.001) and a negative correlation with platelet values (*r* = −0.434, *p* < 0.001).

## 4. Discussion

The aim of this study was to compare the variability of MPV values in hypertension cases. No difference in MPV values was found between the hypertension group and the control group.

The platelet count in peripheral blood was 150−400 × 10^9^/L. They are blood cells with a diameter of 1–3 *μ*m and an MPV of 7–11 fL [11]. Recently, it has been found that the MPV may be used as a biomarker in certain clinical conditions related to inflammation [8, 9, 12].

In hypertension, inflammation occurs in the vascular endothelium. Cytokines secreted in the inflammatory process stimulate megakaryocytes in the bone marrow, and platelet precursors with a high MPV are released into the blood [4, 7, 13].

In a retrospective cohort study, Gang et al. found an increased MPV related to an increase in the incidence of hypertension [14]. In a study by Sileshi et al. examining hematological parameters in hypertensive and normotensive individuals, an MPV of 9.5 ± 2.22 fL was found in hypertensive persons and an MPV 9.04 ± 1.06 fL in normotensive individuals; the difference was statistically significant (*p* = 0.024) [15]. Akın et al. found an MPV of 9.3 ± 1.4 fL in persons with resistant hypertension, an MPV of 8.8 ± 0.8 fL in patients with controlled hypertension, and an MPV of 8.5 ± 0.8 fL in normotensive individuals. The differences between resistant and controlled hypertensive individuals and between resistant and normotensive individuals were statistically significant (*p* = 0.008, *p* < 0.001, respectively) [16]. In a study by Surgit et al., the MPV was 8.97 ± 0.99 fL in individuals with resistant hypertension, 8.46 ± 0.84 fL in patients with controlled hypertension, and 8.17 ± 0.63 fL in normotensive persons; the MPV value was statistically significantly higher in persons with hypertension (*p* < 0.001) [17]. Karabacak et al. found an increase in MPV values during hypertensive crises [18]. In a study by Sansanayudh et al., the MPV was 7.25 ± 0.78 fL in hypertension cases, 7.22 ± 0.78 fL in persons not suffering from hypertension. The difference of MPV values between groups was not statistically significant (*p* = 0.775) [19]. In our study, the MPV was 8.49 ± 0.88 fL in hypertensive individuals and 8.63 ± 0.75 fL in the control group. The difference between the groups was not statistically significant (*p* = 0.405). While most previous studies found a statistically significant elevation of the MPV value in hypertensive patients compared to normotensive individuals, a smaller number of studies, including our own, did not find a statistically significant difference between the two groups. In our view, this issue needs to be examined in more detail through prospective studies with larger case numbers.

Studies by Çetin and Kavaz Tufan applying ambulatory monitoring of arterial pressure compared platelet indices between children with non-dipper and dipper hypertension and normotensive children. The MPV values in children with non-dipper and dipper hypertension were higher than in normotensive children (8.69 ± 0.84, 9.01 ± 1.14, 8.1 ±0 .092, respectively; *p* < 0.05) [20]. In a similar study by Meriç et al., the MPV value in patients with non-dipper and dipper hypertension was higher than in control cases (9.7 ± 1.4, 8.4 ± 0.8, 8.0 ± 0.4, respectively; *p* < 0.001) [21]. As Holter monitoring data for most cases in our study were not available, we cannot comment on this aspect.

While a positive correlation of MPV values with systolic and diastolic arterial pressure has been found [17, 22], our study established no correlation between arterial pressure and MPV.

In hypertensive children, the carotis intima media (CIM) is thicker than in children not suffering from hypertension [23, 24]. Equally, our study found a statistically significant increase in the thickness of the CIM. As hypertension in children can be a significant risk factor for adult atherosclerosis, it may be assumed that monitoring the thickness of the CIM could be relevant.

Hypertension can lead to echocardiographic changes in cardiac indicators [25, 26]. In line with other work, our study demonstrated a statistically significant difference in echocardiographic variability between hypertensive individuals and the control group.

Limitations of our study include a relatively small number of cases compared to other studies and the retrospective design; 24-hour ambulatory monitoring of arterial pressure was not carried out for all cases; and factors affecting hypertension were not evaluated together. On the other hand, while the comparison of MPV values between hypertensive cases and a control group is better studied in adulthood, data about childhood are fairly limited. Repeating this study with a prospective design, using higher case numbers and evaluating factors affecting hypertension as a whole will increase the significance of the results.

## 5. Conclusion

While MPV is hypothesized to be higher in hypertensive individuals, there was no statistically significant difference between the hypertensive and non-hypertensive groups in this study. However, we think that it would be appropriate to reevaluate this issue with prospective studies that include greater numbers of cases. The thickness of the left ventricular wall and carotid intima were shown to be greater in hypertensive individuals in this study, as was expected [2, 27].

## Figures and Tables

**Table 1:** Demographic characteristics of the cases.

	Hypertension group (*n* = 45)	Control group (*n* = 45)	*p* value
Gender			
Female, *n* (%)	19 (42.2)	27 (60)	0.092^*∗*^
Male, *n* (%)	26 (57.8)	18 (40)	
Age (years)			
Median (25p–75p)	15 (13–16)	13 (11–16)	0.104^&^
Weight (kg ± SD)	84.4 ± 17.5	46.8 ± 14.6	**<0.001** ^**#**^
Height (cm ± SD)	169 ± 10.4	156 ± 17.2	**<0.001** ^**#**^
Body mass index (kg/m^2^ ± SD)	29.7 ± 6.20	18.9 ± 2.82	**<0.001** ^**#**^
Systolic blood pressure (mmHg) median (25p–75p)	150 (140–160)	100 (97–110)	**<0.001** ^**&**^
Diastolic blood pressure (mmHg) median (25p–75p)	90 (80–90)	60 (60–65)	**<0.001** ^**&**^

^*∗*^Chi-square test, &Mann-Whitney U test, #Student's t-test. The bold values represent statistically significant *p* values (*p* < 0.05).

**Table 2:** Carotid intima-media thickness and echocardiographic findings of the cases.

	Hypertension group			Control group			*p* value
	*n*	Mean ± SD	Median (25p–75p)	*n*	Mean ± SD	Median (25p–75p)	
CIMT (mm)	43	0.49 ± 0.08	0.50 (0.40–0.50)	26	0.39 ± 0.06	0.40 (0.31–0.40)	**<0.001** ^*∗*^
IVSd (cm)	43	0.78 ± 0.11	0.75 (0.71–0.82)	25	0.61 ± 0.11	0.58 (0.51–0.69)	**<0.001** ^*∗*^
LVDd (cm)	43	4.89 ± 0.41	4.83 (4.54–5.20)	25	4.38 ± 0.58	4.25 (3.99–4.70)	**<0.001** ^*∗*^
LVPWd (cm)	43	0.77 ± 0.12	0.75 (0.70–0.85)	25	0.61 ± 0.11	0.60 (0.56–0.64)	**<0.001** ^*∗*^
IVSs (cm)	39	1.07 ± 0.19	1.00 (0.90–1.19)	18	0.83 ± 0.13	0.85 (0.76–0.92)	**<0.001** ^*∗*^
LVDs (cm)	41	3.21 ± 0.35	3.2 (2.92–3.40)	24	2.86 ± 0.41	2.84 (2.52–3.18)	**<0.001** ^**#**^
LVPWs (cm)	32	1.14 ± 0.14	1.20 (1.00–1.20)	21	0.91 ± 0.13	0.90 (0.80–1.00)	**<0.001** ^*∗*^
EF (%)	41	62.6 ± 6.86	60 (60–65)	25	64.4 ± 4.20	64 (60–68)	0.170^*∗*^
FS (%)	41	33.5 ± 4.15	33 (30–36)	25	34.3 ± 3.80	34 (30–37)	0.254^*∗*^
LVmass (g)	42	128 ± 28.3	123 (106–146)	25	79.6 ± 30.6	70.8 (55.3–106)	**<0.001** ^*∗*^
Mitral-E (m/sn)	36	0.91 ± 0.16	0.90 (0.80–0.99)	26	0.91 ± 0.09	0.91 (0.85–0.97)	0.905^#^
Mitral-A (m/sn)	36	0.56 ± 0.11	0.54 (0.50–0.60)	26	0.54 ± 0.12	0.52 (0.46–0.60)	0.236^*∗*^
Mitral E/A	36	1.67 ± 0.37	1.71 (1.38–1.82)	26	1.75 ± 0.32	1.67 (1.54–1.95)	0.406^#^

CIMT: carotis intima-media thickness, IVSd: interventricular septal thickness at end-diastole, LVDd: left ventricular dimension end-diastolic diameter, LVPWd: left ventricular posterior wall thickness in end-diastole, IVSs: interventricular septal thickness at end-systole, LVDs: left ventricular dimension end-systolic diameter, LVPWs: left ventricular posterior wall thickness in end-systole EF: ejection fraction, FS: fractional shortening, LVmass: left ventricular mass, mitral-E: early diastolic velocity, mitral-A: late diastolic velocity, mitral E/A: mitral peak E/A wave velocity ratio. ^*∗*^Mann–Whitney *U* test, ^#^Student's *t*-test. The bold values represent statistically significant *p* values (*p* < 0.05).

**Table 3:** Complete blood count findings of the cases.

	Hypertension group (*n* = 45)		Control group (*n* = 45)		*p* value
	Mean ± SD	Median (25p–75p)	Mean ± SD	Median (25p–75p)	
RBC (10^6^/*μ*L)	4.89 ± 0.54	4.87 (4.50–5.19)	4.61 ± 0.43	4.62 (4.31–5.00)	**0.009** ^**#**^
Hb (g/dL)	13.7 ± 1.59	13.7 (12.4–15.1)	13.0 ± 1.41	12.6 (12.1–13.7)	**0.032** ^**#**^
Htc (%)	41.7 ± 4.67	40.8 (38.3–45.1)	39.2 ± 3.96	38.2 (36.4–42.1)	**0.012** ^*∗*^
MCV (fL)	85.2 ± 5.38	85.0 (82.0–89.0)	85.3 ± 7.04	85.0 (82.0–90.0)	0.616^*∗*^
MCH (pg)	28.1 ± 1.94	28.3 (27.3–29.2)	28.3 ± 2.67	28.3 (26.9–30.1)	0.347^*∗*^
MCHC (g/dL)	33.0 ± 1.10	33.2 (32.2–33.7)	33.2 ± 0.94	33.1 (32.9–33.8)	0.399^#^
RDW (%)	14.9 ± 1.88	14.5 (13.8–16.2)	14.2 ± 1.54	14.0 (13.0–14.7)	0.058^*∗*^
Leukocyte (10^3^/*μ*L)	6.98 ± 1.80	6.85 (5.92–7.88)	6.77 ± 1.66	6.50 (5.81–7.69)	0.603^*∗*^
Neutrophil (10^3^/*μ*L)	3.52 ± 1.18	3.29 (2.65–4.07)	3.66 ± 1.23	3.57 (2.99–4.37)	0.458^*∗*^
Lymphocyte (10^3^/*μ*L)	2.71 ± 0.84	2.68 (1.99–3.28)	2.45 ± 0.74	2.41 (1.92–2.80)	0.117^#^
Monocyte (10^3^/*μ*L)	0.53 ± 0.20	0.51 (0.42–0.62)	0.50 ± 0.20	0.48 (0.37–0.57)	0.250^*∗*^
Eosinophil (10^3^/*μ*L)	0.20 ± 0.13	0.15 (0.12–0.25)	0.16 ± 0.08	0.14 (0.10–0.21)	0.349^*∗*^
Basophil (10^3^/*μ*L)	0.03 ± 0.02	0.02 (0.01–0.04)	0.03 ± 0.02	0.02 (0.02–0.03)	0.533^*∗*^
Platelets (10^3^/*μ*L)	295.78 ± 73.78	292 (236–357)	275.49 ± 58.29	281 (230–311)	0.151^#^
MPV (fL)	8.49 ± 0.88	8.60 (7.80–9.10)	8.63 ± 0.75	8.60 (8.20–9.10)	0.405^#^
PDW (%)	14.3 ± 2.31	13.8 (12.5–16.0)	14.5 ± 3.09	14.5 (13.5–15.8)	0.214^*∗*^

RBC: red blood cells, Hb: hemoglobin, Htc: hematocrit, MCV: mean corpuscular volume, MCH: mean corpuscular hemoglobin, MCHC: mean corpuscular hemoglobin concentration, RDW: red cell distribution width, MPV: mean platelet volume, PDW: platelet distribution width ^*∗*^Mann-Whitney *U* test, ^#^Student's *t*-test. The bold values represent statistically significant *p* values (*p* < 0.05).

## Data Availability

The data used to support the findings of this study are available from the corresponding author upon reasonable request.
